# Early transcriptional response to gravistimulation in poplar without phototropic confounding factors

**DOI:** 10.1093/aobpla/plaa071

**Published:** 2020-12-31

**Authors:** David Lopez, Jérôme Franchel, Jean-Stéphane Venisse, Joël R Drevet, Philippe Label, Catherine Coutand, Patricia Roeckel-Drevet

**Affiliations:** 1 CIRAD, UMR AGAP, Montpellier, France; 2 AGAP, Univ Montpellier, CIRAD, INRAE, Institut Agro, Montpellier, France; 3 Université Clermont Auvergne, INRAE, PIAF, Campus Universitaire des Cézeaux, 1 Impasse Amélie Murat, TSA, Aubière Cedex, France; 4 Université Clermont Auvergne, GReD INSERM U1103-CNRS UMR 6293, Faculté de Médecine, CRBC (Centre de Recherche Bio-Clinique), Clermont-Ferrand, France; 5 INRAE, UR 115 PSH, Centre de recherche PACA, 228, route de l’aérodrome, CS, Avignon Cedex, France

**Keywords:** Cell wall, Fluidigm, gravitropism, gravity, isotropic light, phototropism, *Populus alba*, *Populus tremula*, qPCR, tension wood, transcriptome, xylem poplar

## Abstract

In response to gravistimulation under anisotropic light, tree stems showing an active cambium produce reaction wood that redirects the axis of the trees. Several studies have described transcriptomic or proteomic models of reaction wood relative to the opposite wood. However, the mechanisms leading to the formation of reaction wood are difficult to decipher because so many environmental factors can induce various signalling pathways leading to this developmental reprogramming. Using an innovative isotropic device where the phototropic response does not interfere with gravistimulation we characterized the early molecular responses occurring in the stem of poplar after gravistimulation in an isotropic environment, and without deformation of the stem. After 30 min tilting at 35° under anisotropic light, we collected the upper and lower xylems from the inclined stems. Controls were collected from vertical stems. We used a microarray approach to identify differentially expressed transcripts. High-throughput real-time PCR allowed a kinetic experiment at 0, 30, 120 and 180 min after tilting at 35°, with candidate genes. We identified 668 differentially expressed transcripts, from which we selected 153 candidates for additional Fluidigm qPCR assessment. Five candidate co-expression gene clusters have been identified after the kinetic monitoring of the expression of candidate genes. Gene ontology analyses indicate that molecular reprogramming of processes such as ‘wood cell expansion’, ‘cell wall reorganization’ and ‘programmed cell death’ occur as early as 30 min after gravistimulation. Of note is that the change in the expression of different genes involves a fine regulation of gibberellin and brassinosteroid pathways as well as flavonoid and phosphoinositide pathways. Our experimental set-up allowed the identification of genes regulated in early gravitropic response without the bias introduced by phototropic and stem bending responses.

## Teaser

In a heterogeneous stand, the individual tree may be subjected to an anisotropic light environment causing phototropic reactions that will lead to gravitropic movements. The reorientation of the tree is due to the production of asymmetric reaction wood that affects the shape of the trunk and the quality of the wood. The molecular events leading to the formation of reaction wood are complex. By tilting young poplars 30 min at 35° in an innovative isotropic light device, we have highlighted a list of genes that respond specifically to the gravitropic stimulus.

## Introduction

The perception of Earth’s gravity leads to a gravity-driven growth process called gravitropism. In higher plants, shoots grow upwards (negative gravitropism), while roots grow downwards (positive gravitropism). Gravitropism includes the perception of gravity, signal transmission and growth response. Many studies were conducted on roots in which the key gravity detection site (columella cells at the root tip) is separated from the bending zone (extension zone) (for a review of the topic, see Baldwin *et al.* 2013). Several plant species were also used to study the response to gravistimulation of aerial parts including maize coleoptiles, pulvini barley, *Pisum* hypocotyls, *Arabidopsis* inflorescence stems and hypocotyls (for a review, see Hashiguchi *et al.* 2013). In these herbaceous species, vertical root or shoot movements are caused by differential elongation between the upper and lower parts of the gravistimulated organs. In trees, two different motors allow the curvature of the stem as reorientation is obtained by differential growth in areas of primary elongation, while it is obtained by differential cellular differentiation between the two sides of the stem in areas with active cambium (for a review, see Groover 2016). Consequently, the cambium produces an asymmetric ‘reaction wood’ (Côté and Day 1964). In gymnosperms, ‘reaction wood’ is called ‘compression wood’ and develops on the underside of the inclined rods where it generates a compressive force to push the stem up (Timell 1986). In arboreal dicotyledonous angiosperms, the ‘reaction wood’ is called ‘tension wood’ (TW) and forms on the upper face of the inclined stem when the tree begins the reorientation process (for a review, see Du and Yamamamoto 2007). The ‘opposite wood’ forms on the underside and is anatomically similar to the ‘normal wood’ formed by straight stems. Tension wood is a particular type of secondary xylem that generates high internal tension forces, mainly due to the longitudinal shrinkage of maturing wood cells (Kubler 1959). Ruelle (2014) reviewed the variations in the anatomy and ultrastructure of TW compared to the ‘opposite wood’ (opposite side of the stem) and ‘normal wood’ (in a non-tilted tree). In many angiosperms, TW is characterized by the presence of gelatinous fibres (G-layer) of almost pure cellulose.

The molecular basis of the early events that followed gravistimulation and led to the formation of TW is still fragmentary. The literature review showed that most studies using comprehensive approaches such as proteomics and transcriptomics have examined the formation of ‘reaction wood’ at developmental stages when it is already histologically observable and/or when the tree undergoes recovery movement (for a review, see Tocquard *et al.* 2014). For example, Andersson-Gunneras *et al*. (2006) pioneering work reported a complete quantification of gene expression and related metabolites occurring during TW formation in poplar. In this study, xylem samples from plants that had been tilted for 3 weeks were used, focussing the analysis on the stage of the secondary wall formation. Chen *et al.* (2015) characterized gene expression patterns, in TW, opposite and normal wood in 30-year-old *Populus tomentosa*. Gerttula *et al.* (2015) produced genomic transcriptomes for 3-month-old *Populus tremula × alba*, stimulated either by gravistimulation (placed horizontally for 2 days), gibberellic acid treatment or ARBORKNOX2 expression.

In the present work, we have focussed on deciphering transcriptomic events caused by stem tilting before the beginning of the righting movement. For that reason, xylem samples were collected after 30 min of tilting, before the appearance of gravitational motion and TW formation. In addition, to our knowledge, previous experimental designs did not distinguish gravisensing from mechanosensing signalling pathways (Lopez *et al.* 2014; Groover 2016). For example, although Azri *et al*. (2009, 2013) have characterized the proteome of young poplars inclined at 35° for 45 min. In that particular set-up, the inclined organ induces flexion under its own weight, which can be considered as a thigmomorphogenetic stimulus (Coutand 2010). To avoid such situation, we limited our analyses to the part of the stem showing secondary growth. The trees were planted a week before use, so there was no bending due to the weight of the tree after tipping. Finally, and most importantly, phototropic and gravitropic responses as well as the autotropic response, which is essentially the straightening of a curved organ, are processes in constant interaction under natural conditions that were not dissociated in the experimental situations reported so far (Bastien *et al.* 2015).

In a recent study, we succeeded in dissociating these complex stimuli thanks to an original device that allowed us to combine isotropic lighting and tilting of young poplars (Coutand *et al.* 2019), thus avoiding the phototropic response. In such a device, we showed that the gravitropic response was partly distinct when comparing an anisotropic light condition to an isotropic light condition (Coutand *et al.* 2019). To better understand the molecular basis of these responses, we used here this device that allows us to distinguish between gravitropic, mechano-sensitive and phototropic responses in plants in order to study the molecular adjustments due to gravistimulation on stems in the early phases of ‘reaction wood’ formation.

## Materials and Methods

### Plant material

The study was conducted on young poplars (*Populus tremula × Populus alba* cv 717 1B4) produced by *in vitro* propagation (Azri *et al.* 2009). They were placed in growth chambers after acclimation (photoperiod: 16 h/8 h; day: 24 °C, night: 18 °C). For transcriptome studies, plants with straight stem, aged 3 months, with a height of between 40 and 50 cm and an average diameter at half height of 10 mm were carefully and loosely staked avoiding any mechanical stress 1 week before use. The side of the stem to the stake was marked with dots using a black marker.

### Isotropic light device and tilting of the plants

**Figure 1.**
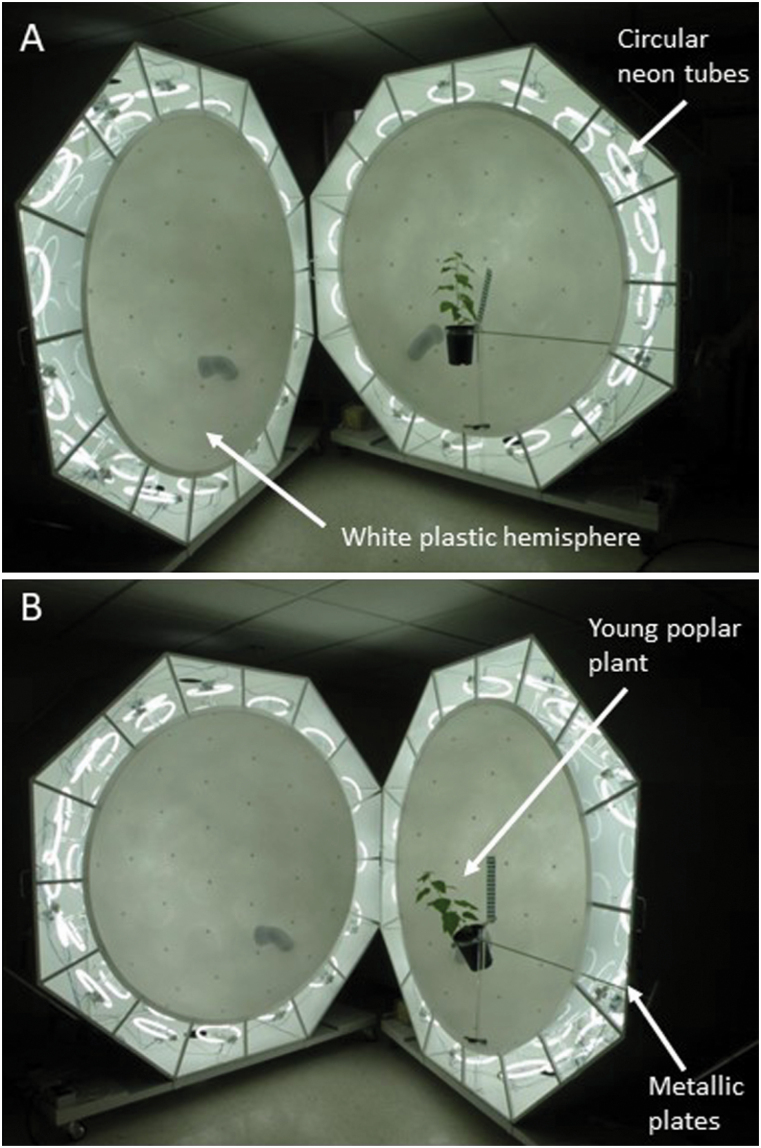
Experimental device of isotropic light to disentangle gravitropic and phototropic plant responses. The isotropic light is obtained from circular neon tubes arranged in a turtle pattern at the periphery of the white plastic hemisphere. The light is concentrated towards the inside of the hemisphere by metallic plates covering the device. The poplar plant is placed in the centre of the device, and the hemispheres are tightly connected (Coutand *et al.* 2019 for the full description of the isotropic device). (A) Straight young poplar plant. (B) Plant subjected to tilting.

The spheres used for gravistimulation were 150 cm wide and uniformly bright (i.e. isotropic light; Coutand *et al.* 2019) (Fig. 1). Growing conditions were 16 h/8 h photoperiod; 24 °C (day)/18 °C (night). The temperature was maintained by a controlled cooling system (blyss, WAP 267EC).

**Figure 2.**
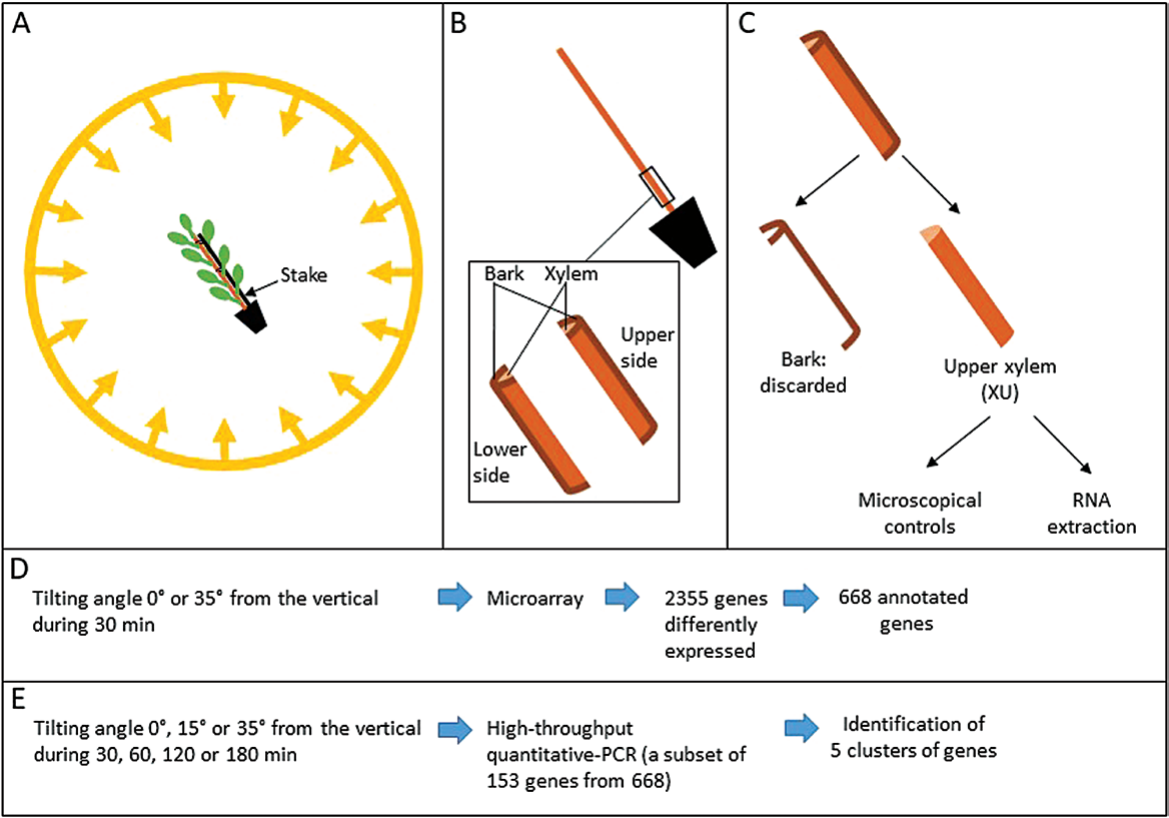
Scheme of the procedures. (A) Isotropic device containing a stalked plant. (B) Longitudinal splitting of the stem. (C) Isolation of XU samples used for RNA extraction and microscopical controls. (D) Microarray experiment. (E) Candidate gene exploration.

One week after staking (staking took place in the growth chamber), the plants reached 55 cm (SD = 3.7 cm, *n* = 76). Staked trees were placed in the sphere (one plant *per* sphere). First of all, the plants were left in an upright position for 24 h. Then, 7 h after the beginning of the photoperiod, the plants were tilted 35° from the vertical. The control plants were kept straight in the sphere. When tilted, the stake was oriented beside the upper face of the stem to prevent the stem from bending due to its own weight (Fig. 2A).

### Tissue sampling

After treatment, for each plant, we selected a stem segment (15 cm long, 5 cm above ground level, i.e. internodes 4–11) that did not contain any TW according to Astra-blue/Safranine staining (Azri *et al.* 2009). In this part of the stem, the cambium is active and a secondary xylem is formed. Stem segments 0.5 mm long were taken from internodes 4, 7 and 11 for further microscopic checks. The rest of the stem was divided longitudinally into two parts using a scalpel (Fig. 2B). The lower (opposite the stake) and upper (towards the stake) sides of the stems were collected; the bark was peeled and discarded (Fig. 2C). Lower xylem (XL) and upper xylem (XU) were instantly frozen in liquid nitrogen and stored. We used the same protocol for straight stems (controls). The xylem to the stake was used for the comparison with XU, while the xylem opposite the stake was used for the comparison with XL.

### RNA extraction

When no TW was detected in the stem by microscopic controls, the xylem samples were crushed in liquid nitrogen. The total RNA was extracted from XU and XL samples and controls (also arbitrarily referred to as XU and XL) using the Qiagen RNaeasy Plant Kit according to the supplier’s instructions (Qiagen, Courtaboeuf, France).

### Transcriptome studies using DNA microarray technology

The discovery of genes whose expression has been modulated by inclination has been carried out using a microarray approach. RNA samples were obtained from the upper or lower xylem of four inclined plants (30 min, 35°) and four straight plants. For each comparison, a technical repeat with fluorochrome inversion including four biological repetitions was performed (i.e. 4 dye-switch hybridizations per comparison).

The hybridization, scanning and analysis of the biochips were performed by the Plant Genomics Resarch Unit (Evry, France) according to their standard procedures. From 200 ng total RNA, amplification and labelling of cDNA with Cy3-dUTP or Cy5-dUTP (Perkin-Elmer-NEN Life Science Products) was performed with the TransPlex Complete Whole Transcriptome Amplification Kit (WT2A; Sigma-Aldrich, Inc.) as recommended by the manufacturer. The *Populus* microarray based on Roche-NimbleGen technology (Roche, Boulogne-Billancourt, France) was used. A single high-density *Populus* microarray slide contains 12 chambers, each containing 135 029 primers representing 49 474 gene models based on the annotation of the *Populus trichocarpa* v1.1 genome. Hybridization and washing were carried out in accordance with the recommendations of NimbleGen (Roche NimbleGenR Technologies, Inc.). A two-micron scan was performed with the InnoScan900 scanner (InnopsysR, Carbonne, France) and the raw data were extracted with the MapixR software (InnopsysR, Carbonne, France). The measured fold variation was corrected according to Bonferroni, followed by a Benjamini–Hochberg *P*-value adjustment (Benjamini and Hochberg 2000). Biochip data from this study have been deposited at the Gene Expression Omnibus (http://www.ncbi.nlm.nih.gov/geo/, access number GSE86951) and CATdb (http://urgv.evry.inra.fr/CATdb/; Project: 12plex-poplar-2012_01) according to MIAME standards.

### Statistical analysis of microarray data

For each array, the raw data included the logarithm of the median pixel intensity of the characteristics at 635 nm (red) and 532 nm (green) wavelengths. For each array, global normalization according to intensity was performed using the LOESS procedure (Yang *et al.* 2002) to correct for dye bias. The differential analysis was based on the average of log-ratios on duplicate probes and technical repetitions. Therefore, the number of data available for each gene is equal to the number of biological replicates and was used to calculate the moderate *t*-test (Smyth 2004). Under the null hypothesis, Limma (version 3.34.8) found no evidence that specific variances varied from one probe to another and, therefore, the moderate *t*-statistics was assumed to follow a standard normal distribution. To control the rate of false discoveries, the corrected *P*-values found were calculated using the optimized FDR approach (Storey 2003). We considered the probes with an adjusted *P*-value ≤ 0.05 to be differentially expressed.

The analysis was done with the R software (version 3.4.3, R Core Team (2017). R: A language and environment for statistical calculation. R Foundation for Statistical Computing, Vienna, Austria. https://www.R-project.org/.). The SqueezeVar function of the Limma library was used to smooth specific variances by calculating empirical Bayesian posterior averages. The library kerfdr (version 2.0.1) was used to calculate the adjusted *P*-values.

Data management, statistical analysis and hierarchical grouping were performed using R. Hierarchical grouping was calculated using the hclust function (set of statistics) using Euclidean distances and the Ward method. The false discovery rate was calculated using the p.adjust function (set of statistics) using the Benjamini and Yekutieli (2001) method.

### Validation of microarray results

For the qPCR validation, we selected 153 genes amongst the most differentially expressed genes from biochip analyses. Before proceeding with the qPCR analysis, the cDNA was synthesized from 0.5 µg total RNA using SuperScript III (Invitrogen, USA) with a 1:1 mixture of oligo-dT and random hexamers. The primer pairs were designed using the Quantprime online service (www.quantprime.de) **[see**  **Supporting Information—Table S1****]**. The specificity of the primers was ensured by checking the melting curve and sequencing the PCR products on the cDNA. The primers were validated when the efficiency was close to 100 % on the slopes of the standard curve. Relative quantification with respect to vertical controls was performed using the 2^−∆∆Cq^ method developed by Pfaffl (2001). The expression levels of the 153 genes obtained by qPCR and microarray analysis were compared using an analysis of variance followed by a Tukey Honest Significance Difference (HSD) *post hoc* test.

### Kinetics study of candidate gene expression

The plants were either straight or inclined at 35° for 30, 60, 120 or 180 min in the isotropic device. Eight independent biological replicates (individual plants) were produced for each condition. XU and XL cDNA samples were used to monitor the expression of the 153 candidate genes by real-time qPCR at high-throughput. The experiments were performed according to the Fluidigm gene expression protocol without modification using the Biomark platform (Fluidigm, USA) and the 96x96 IFC gene expression (integrated fluidic circuit). Relative quantification was performed using the 2^−∆∆Cq^ method developed by Pfaffl (2001). Expression levels were related to the geometric mean of the 153 expression levels of the candidate genes proposed by the Bestkeeper reference research software (Vandesompele *et al.* 2002; Pfaffl *et al.* 2004), but using a larger subset of genes. No significant Tukey *post hoc* HSD was found in the change in gene expression (*P* < 0.05, data not shown) between qPCR and microarray, which validated the microarray results.

### Bioinformatics analyses

The functional annotation was retrieved using a standalone version of the BioMart software (http://biomart.org) and the genomic annotation *P. trichocarpa* v3.0 (http://phytozome.jgi.doe.gov). The singular AgriGO enrichment analysis (http://bioinfo.cau.edu.cn/agriGO/, Zhou *et al.* 2010) was used for the analysis of gene set enrichment (GSEA) using the genetic ontology terms (GO) annotated for genes identified on the DNA chip and the default AgriGO parameters, adapted to large data sets (Fisher test, Yekutieli multiple test adjustment method and significance level at 0.05). We evaluated the possible bias of the GO term enrichment procedure when using the Nimblegene poplar network by comparing the whole gene models, based on *P. trichocarpa* V1.1, present on the Nimblegene array with the *P. trichocarpa* V3 genome gene models. No significant representativeness bias was detected. Thus, we considered that the enrichment of the term GO based on Nimblegene data was valid when using GO annotations of the poplar genome V3. Further analyses were carried out using PopGenIE (http://popgenie.org, Sjödin *et al.* 2009; Sundell *et al.* 2015) for the analysis of qPCR clusters and the discovery of miRNA targets.

## Results

### Neutral discovery of early modulated genes

In the isotropic device, the phototropic response cannot occur. This device was used to identify genes regulated by gravistimulation in forming ‘reaction wood’ using DNA chip technology (Nimblegene microarray). Analyses of xylem samples were carried out by comparing the expression levels of vertical (control) and inclined plants in the lower part of the xylem (XL, i.e. the face facing the ground when it is inclined) and in the upper part of the xylem (XU). Array hybridizations resulted in a subset of 2355 gene models (poplar genome version 1.1) expressed differentially after Benjamini and Hochberg as well as Bonferroni multiple test corrections were made (*P*-value < 0.05; **see**  **Supporting Information—Table S2**). Our experimental set-up provided well-controlled conditions, as evidenced by the reproducibility of the microarray experiments **[see**  **Supporting Information—Fig. S1****]**.

**Figure 3.**
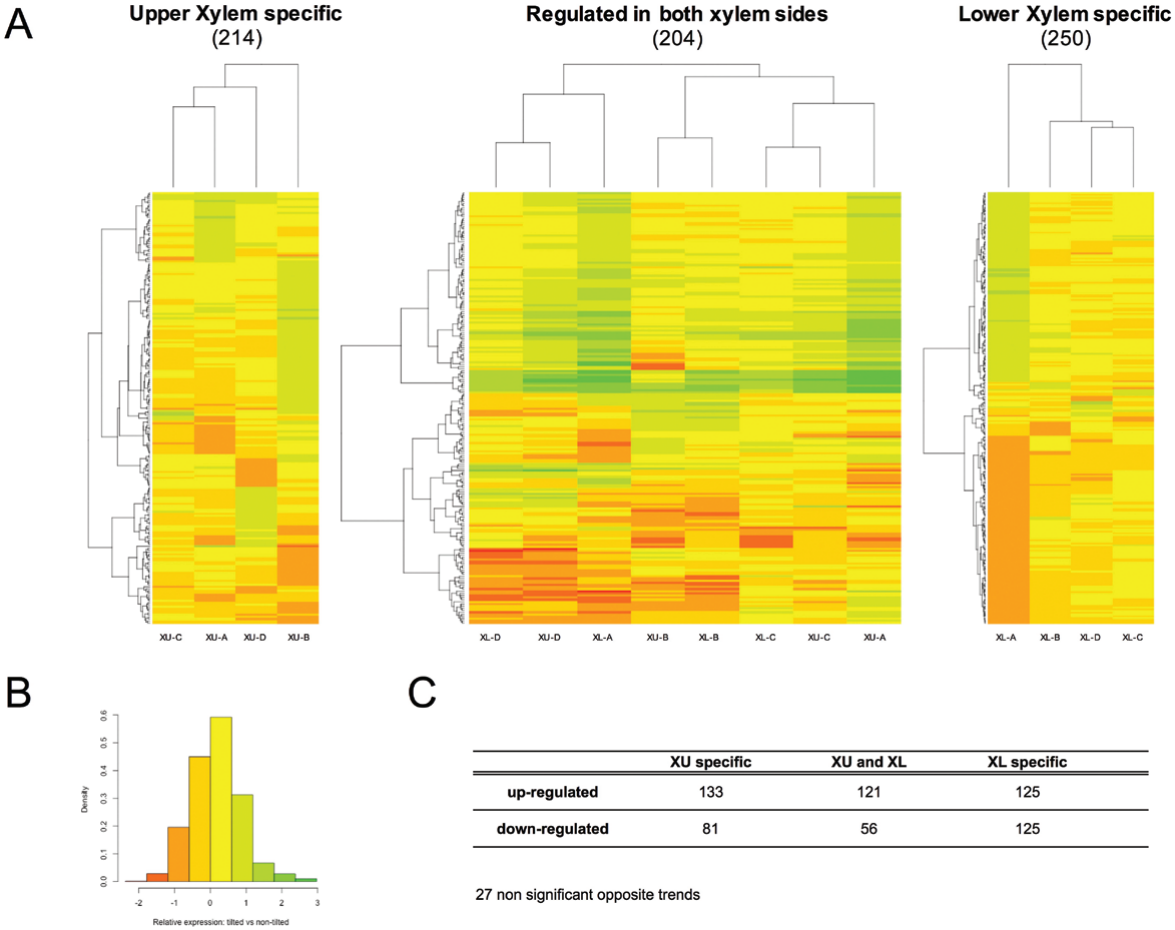
Identification of poplar genes with expression significantly regulated in response to a 30min, 35° gravi-stimulation in xylem tissues. (A) Heatmaps of differentially expressed genes found in Upper Xylem (XU, 214), Lower Xylem (XL) and in both XU and XL. Gene models are hierarchically clustered on the Y-axis and the four biological repeats are hierarchically clustered on the X-axis. (B) Key of the heatmaps, fold changes are represented on the X-axis and frequencies on the Y-axis. (C) Detail by tissue and trend of the gene expression patterns. Note that 27 genes showed a significant regulation in both XU and XL but with opposite trends. However, none of these “opposite” trends were statistically significant hence, they are not accounted in the “XU and XL” column.

From the list of 2355 genes, 668 showed significant differential expression between the two conditions (annotation was performed using the poplar genome version 3; **see**  **Supporting Information—Table S3**). Of these, 214 and 250 genes were modulated exclusively in XU or XL, respectively, while 204 were modulated on both sides of the xylem in response to gravity (Fig. 3A). For this last set of genes, there is no tendency for ‘side-specific’ expression, as confirmed by the hierarchical grouping of biological repetitions showing that the XL and XU samples are mixed. A high-resolution version of the heatmaps containing the names of the gene models is available in **Supporting Information—Fig. S2**. As shown in the expression maps and density histogram (Figs 3A and B), the most differentially expressed genes are within a range of 2-fold variation between inclined and control trees. Figure 3C shows, a total of 379 genes were always regulated upward (133 + 121 + 125) and 262 genes (81 + 56 + 125) were always regulated downward.

### Functional exploration

In order to clarify the role of modulated genes in response to gravistimulation, the GSEA was performed using GO term enrichment with the three different ontologies available (Biological Process (BP); Molecular Function (MF); Cell Component (CC)) and calculated with AgriGO. Considering the 668 genes significantly differentially expressed for GSEA, 104 GO terms had a significantly different frequency of occurrence and a wide range of BPs were affected (Table 1; **see**  **Supporting Information—Fig. S3**). Among the 67 significant BP terms brought forward, ‘hormone (ethylene and brassinosteroids) level response and regulation’, ‘cell wall biogenesis’, ‘amino acid metabolism’, ‘phenylpropanoid metabolism’, ‘lignin catabolic process’, ‘carbohydrate metabolism’ and ‘microtubule-based movements’ were the most prominent. For the MF terms, ‘ion binding’ was the most over-represented term, followed by ‘oxidoreductase activity’ and ‘transmembrane transport’. Several CC terms of interest were affected including ‘membrane and endomembrane systems’, ‘cell wall’, ‘apoplast’, ‘amyloplast’, ‘vacuole’ and ‘tubulin complex’ **[see**  **Supporting Information—Fig. S3****]**.

**Table 1.** GO term enrichment analysis of the 668 differentially expressed xylem genes (35° tilted vs. control).

GO term	Ontology	Description	Number in input list	Number in BG/Ref	*P*-value	FDR	GO term	Ontology	Description	Number in input list	Number in BG/Ref	*P*-value	FDR
GO:0005975	P	Carbohydrate metabolic process	71	2018	5.10E-10	1.50E-06	GO:0046271	P	Phenylpropanoid catabolic process	5	37	0.00046	0.025
GO:0034641	P	Cellular nitrogen compound metabolic process	45	1031	2.40E-09	2.30E-06	GO:0042180	P	Cellular ketone metabolic process	49	1886	0.00056	0.03
GO:0005976	P	Polysaccharide metabolic process	34	639	2.40E-09	2.30E-06	GO:0070297	P	Regulation of two-component signal transduction system (phosphorelay)	5	40	0.00064	0.032
GO:0044264	P	Cellular polysaccharide metabolic process	26	457	5.30E-08	3.80E-05	GO:0010104	P	Regulation of ethylene-mediated signalling pathway	5	40	0.00064	0.032
GO:0016051	P	Carbohydrate biosynthetic process	31	641	9.40E-08	5.40E-05	GO:0010105	P	Negative regulation of ethylene-mediated signalling pathway	5	40	0.00064	0.032
GO:0006519	P	Cellular amino acid and derivative metabolic process	57	1688	1.10E-07	5.40E-05	GO:0070298	P	Negative regulation of two-component signal transduction system (phosphorelay)	5	40	0.00064	0.032
GO:0006073	P	Cellular glucan metabolic process	20	298	1.60E-07	6.50E-05	GO:0009063	P	Cellular amino acid catabolic process	9	144	0.00067	0.033
GO:0044262	P	Cellular carbohydrate metabolic process	41	1093	6.10E-07	0.00022	GO:0009266	P	Response to temperature stimulus	35	1229	0.00077	0.037
GO:0044042	P	Glucan metabolic process	20	329	7.00E-07	0.00022	GO:0009310	P	Amine catabolic process	9	149	0.00084	0.04
GO:0019748	P	Secondary metabolic process	44	1235	8.90E-07	0.00026	GO:0022611	P	Dormancy process	5	43	0.00087	0.04
GO:0009066	P	Aspartate family amino acid metabolic process	13	145	1.20E-06	0.00032	GO:0046395	P	Carboxylic acid catabolic process	12	253	0.00097	0.043
GO:0034637	P	Cellular carbohydrate biosynthetic process	24	483	1.70E-06	0.00037	GO:0000097	P	Sulfur amino acid biosynthetic process	6	67	0.00095	0.043
GO:0007018	P	Microtubule-based movement	14	174	1.60E-06	0.00037	GO:0016054	P	Organic acid catabolic process	12	253	0.00097	0.043
GO:0009308	P	Amine metabolic process	41	1171	3.20E-06	0.00061	GO:0009873	P	Ethylene-mediated signalling pathway	10	185	0.00099	0.043
GO:0006555	P	Methionine metabolic process	10	88	3.00E-06	0.00061	GO:0009312	P	Oligosaccharide biosynthetic process	7	95	0.0011	0.046
GO:0033692	P	Cellular polysaccharide biosynthetic process	18	311	4.70E-06	0.00085	GO:0016491	F	Oxidoreductase activity	97	3249	1.30E-09	1.30E-06
GO:0005978	P	Glycogen biosynthetic process	6	24	5.80E-06	0.00098	GO:0005506	F	Iron ion binding	49	1268	1.90E-08	9.30E-06
GO:0000271	P	Polysaccharide biosynthetic process	19	354	7.20E-06	0.0012	GO:0046906	F	Tetrapyrrole binding	34	819	6.80E-07	0.00022
GO:0044106	P	Cellular amine metabolic process	33	890	9.70E-06	0.0015	GO:0020037	F	Heme binding	31	744	2.00E-06	0.00047
GO:0006575	P	Cellular amino acid derivative metabolic process	33	908	1.40E-05	0.0021	GO:0016638	F	Oxidoreductase activity, acting on the CH-NH_2_ group of donors	10	94	5.20E-06	0.001
GO:0042221	P	Response to chemical stimulus	114	4909	1.50E-05	0.0021	GO:0009055	F	Electron carrier activity	43	1346	1.60E-05	0.0026
GO:0042546	P	Cell wall biogenesis	17	313	1.90E-05	0.0025	GO:0004091	F	Carboxylesterase activity	24	564	2.00E-05	0.0028
GO:0009409	P	Response to cold	31	855	2.70E-05	0.0034	GO:0004553	F	Hydrolase activity, hydrolyzing *O*-glycosyl compounds	29	773	2.70E-05	0.0033
GO:0009415	P	Response to water	24	584	3.40E-05	0.004	GO:0016758	F	Transferase activity, transferring hexosyl groups	27	707	3.80E-05	0.0037
GO:0005977	P	Glycogen metabolic process	7	52	3.50E-05	0.004	GO:0046914	F	Transition metal ion binding	101	4311	3.80E-05	0.0037
GO:0006112	P	Energy reserve metabolic process	7	54	4.40E-05	0.0046	GO:0004497	F	Monooxygenase activity	25	638	5.00E-05	0.004
GO:0000096	P	Sulfur amino acid metabolic process	10	122	4.20E-05	0.0046	GO:0019825	F	Oxygen binding	19	409	4.80E-05	0.004
GO:0009067	P	Aspartate family amino acid biosynthetic process	8	75	4.50E-05	0.0046	GO:0008509	F	Anion transmembrane transporter activity	14	251	7.70E-05	0.0057
GO:0009698	P	Phenylpropanoid metabolic process	25	639	5.10E-05	0.005	GO:0016762	F	Xyloglucan:xyloglucosyl transferase activity	6	44	0.00012	0.0082
GO:0006520	P	Cellular amino acid metabolic process	31	890	5.60E-05	0.0053	GO:0016757	F	Transferase activity, transferring glycosyl groups	31	954	0.00019	0.011
GO:0009250	P	Glucan biosynthetic process	12	184	6.20E-05	0.0057	GO:0016798	F	Hydrolase activity, acting on glycosyl bonds	29	864	0.00018	0.011
GO:0010817	P	Regulation of hormone levels	18	387	7.40E-05	0.0065	GO:0003824	F	Catalytic activity	344	19164	0.00023	0.013
GO:0019439	P	Aromatic compound catabolic process	7	59	7.30E-05	0.0065	GO:0005200	F	Structural constituent of cytoskeleton	7	73	0.00025	0.013
GO:0009832	P	Plant-type cell wall biogenesis	14	252	8.00E-05	0.0068	GO:0015149	F	Hexose transmembrane transporter activity	6	59	0.00051	0.026
GO:0009719	P	Response to endogenous stimulus	69	2734	9.60E-05	0.0079	GO:0022891	F	Substrate-specific transmembrane transporter activity	48	1842	0.0006	0.027
GO:0009834	P	Secondary cell wall biogenesis	9	112	0.00012	0.0093	GO:0008471	F	Laccase activity	5	39	0.00058	0.027
GO:0009414	P	Response to water deprivation	22	560	0.00013	0.01	GO:0016641	F	Oxidoreductase activity, acting on the CH-NH_2_ group of donors, oxygen as acceptor	6	63	0.00071	0.031
GO:0042445	P	Hormone metabolic process	13	232	0.00013	0.01	GO:0043169	F	Cation binding	132	6449	0.0008	0.033
GO:0051258	P	Protein polymerization	10	147	0.00018	0.013	GO:0043167	F	Ion binding	132	6472	0.00091	0.037
GO:0009086	P	Methionine biosynthetic process	5	30	0.00019	0.014	GO:0015145	F	Monosaccharide transmembrane transporter activity	6	67	0.00095	0.037
GO:0009628	P	Response to abiotic stimulus	82	3504	0.00023	0.016	GO:0016705	F	Oxidoreductase activity, acting on paired donors, with incorporation or reduction of molecular oxygen	16	415	0.0013	0.047
GO:0010114	P	Response to red light	10	154	0.00026	0.017	GO:0012505	C	Endomembrane system	98	3339	2.50E-09	1.10E-06
GO:0010876	P	Lipid localization	5	32	0.00025	0.017	GO:0009501	C	Amyloplast	7	25	5.00E-07	0.00011
GO:0019252	P	Starch biosynthetic process	5	35	0.00037	0.022	GO:0045298	C	Tubulin complex	7	33	2.50E-06	0.00035
GO:0009741	P	Response to brassinosteroid stimulus	13	258	0.00035	0.022	GO:0005618	C	Cell wall	37	1051	8.60E-06	0.00082
GO:0043436	P	Oxoacid metabolic process	49	1849	0.00037	0.022	GO:0031225	C	Anchored to membrane	23	501	9.50E-06	0.00082
GO:0019752	P	Carboxylic acid metabolic process	49	1849	0.00037	0.022	GO:0048046	C	Apoplast	15	285	8.00E-05	0.0057
GO:0006082	P	Organic acid metabolic process	49	1852	0.00038	0.023	GO:0030312	C	External encapsulating structure	37	1207	0.00014	0.0076
GO:0046274	P	Lignin catabolic process	5	36	0.00041	0.024	GO:0009505	C	Plant-type cell wall	30	896	0.00014	0.0076
GO:0010033	P	Response to organic substance	76	3252	0.00042	0.024	GO:0031224	C	Intrinsic to membrane	96	4289	0.0003	0.014
GO:0009725	P	Response to hormone stimulus	62	2522	0.00043	0.024	GO:0009705	C	Plant-type vacuole membrane	8	113	0.00062	0.024
GO:0050896	P	Response to stimulus	192	9863	0.00045	0.025	GO:0005576	C	Extracellular region	32	1072	0.00061	0.024

Ontology “P” indicates Biological Process, ontology “F” indicates Molecular Function, and ontology “C” indicates Cellular Component. “Number in input list” is the number of occurrence of the Gene Ontology term in the input list (668 genes). “Number in BG/Ref” is the observed occurrence of the Gene Ontology term in the *Populus trichocarpa* genome. *P*-value represent the statistical significance of the enrichment test and FDR is the false discovery rate set to 0.05.

Tables 2 and 3 list the significant GO terms for the 379 genes regulated upwards and the 262 genes regulated downwards, respectively. It is interesting to note that most enriched GO terms are exclusive to genes regulated upwards or downwards. Since 27 genes with opposite tendencies were included in either group, they were analysed separately (Table 4).

**Table 2.** GO term enrichment analysis of the 379 upregulated xylem genes (35° tilted vs. control).

GO term	Ontology	Description	Number in input list	Number in BG/Ref	*P*-value	FDR
GO:0005977	P	Glycogen metabolic process	5	52	0.00016	0.014
GO:0007017	P	Microtubule-based process	16	605	0.00018	0.014
GO:0006112	P	Energy reserve metabolic process	5	54	0.00019	0.015
GO:0009250	P	Glucan biosynthetic process	8	184	0.00036	0.026
GO:0009873	P	Ethylene-mediated signalling pathway	8	185	0.00037	0.026
GO:0009415	P	Response to water	15	584	0.00038	0.026
GO:0009741	P	Response to brassinosteroid stimulus	9	258	0.00074	0.049
GO:0009834	P	Secondary cell wall biogenesis	8	112	1.30E-05	0.0016
GO:0044262	P	Cellular carbohydrate metabolic process	28	1093	1.30E-06	0.00033
GO:0009409	P	Response to cold	26	855	1.40E-07	8.30E-05
GO:0044042	P	Glucan metabolic process	13	329	1.50E-05	0.0016
GO:0005975	P	Carbohydrate metabolic process	48	2018	1.50E-09	2.80E-06
GO:0006519	P	Cellular amino acid and derivative metabolic process	34	1688	1.60E-05	0.0016
GO:0051258	P	Protein polymerization	10	147	1.70E-06	0.00038
GO:0007018	P	Microtubule-based movement	13	174	1.70E-08	1.50E-05
GO:0009266	P	Response to temperature stimulus	27	1229	2.90E-05	0.0029
GO:0034637	P	Cellular carbohydrate biosynthetic process	17	483	3.40E-06	0.00068
GO:0044264	P	Cellular polysaccharide metabolic process	18	457	3.70E-07	0.00017
GO:0006575	P	Cellular amino acid derivative metabolic process	22	908	4.10E-05	0.0038
GO:0034641	P	Cellular nitrogen compound metabolic process	26	1031	4.10E-06	0.00073
GO:0042546	P	Cell wall biogenesis	12	313	4.30E-05	0.0038
GO:0009832	P	Plant-type cell wall biogenesis	12	252	5.40E-06	0.00083
GO:0006073	P	Cellular glucan metabolic process	13	298	5.50E-06	0.00083
GO:0005976	P	Polysaccharide metabolic process	21	639	6.90E-07	0.00022
GO:0000271	P	Polysaccharide biosynthetic process	14	354	7.10E-06	0.00098
GO:0016051	P	Carbohydrate biosynthetic process	21	641	7.30E-07	0.00022
GO:0033692	P	Cellular polysaccharide biosynthetic process	13	311	8.60E-06	0.0011
GO:0016758	F	Transferase activity, transferring hexosyl groups	18	707	0.00011	0.022
GO:0004553	F	Hydrolase activity, hydrolyzing *O*-glycosyl compounds	18	773	0.00032	0.043
GO:0016641	F	Oxidoreductase activity, acting on the CH-NH_2_ group of donors, oxygen as acceptor	5	63	0.00037	0.043
GO:0005507	F	Copper ion binding	10	301	0.00055	0.048
GO:0016491	F	Oxidoreductase activity	48	3249	0.00057	0.048
GO:0005506	F	Iron ion binding	24	1268	0.00068	0.05
GO:0016638	F	Oxidoreductase activity, acting on the CH-NH_2_ group of donors	7	94	3.60E-05	0.011
GO:0005200	F	Structural constituent of cytoskeleton	7	73	7.90E-06	0.0046
GO:0030312	C	External encapsulating structure	24	1207	0.00034	0.014
GO:0016020	C	Membrane	111	9319	0.00065	0.024
GO:0031224	C	Intrinsic to membrane	59	4289	0.00076	0.025
GO:0044425	C	Membrane part	71	5441	0.00087	0.025
GO:0044430	C	Cytoskeletal part	17	790	0.0011	0.029
GO:0031225	C	Anchored to membrane	21	501	1.40E-08	3.40E-06
GO:0012505	C	Endomembrane system	63	3339	2.30E-08	3.40E-06
GO:0005618	C	Cell wall	24	1051	4.50E-05	0.0033
GO:0045298	C	Tubulin complex	7	33	6.30E-08	6.10E-06
GO:0048046	C	Apoplast	11	285	8.40E-05	0.0047
GO:0009505	C	Plant-type cell wall	21	896	9.60E-05	0.0047

Ontology “P” indicates Biological Process, ontology “F” indicates Molecular Function, and ontology “C” indicates Cellular Component. “Number in input list” is the number of occurrence of the Gene Ontology term in the input list (379 genes,). “Number in BG/Ref” is the observed occurrence of the Gene Ontology term in the *Populus trichocarpa* genome. *P*-value represent the statistical significance of the enrichement test and FDR is the false discovery rate set to 0.05.

**Table 3.** GO term enrichment analysis of the 262 downregulated xylem genes (35° tilted vs. control).

GO term	Ontology	Description	Number in input list	Number in BG/Ref	*P*-value	FDR
GO:0010876	P	Lipid localization	5	32	2.80E-06	0.0037
GO:0009067	P	Aspartate family amino acid biosynthetic process	6	75	9.70E-06	0.0065
GO:0034641	P	Cellular nitrogen compound metabolic process	19	1031	2.20E-05	0.0088
GO:0042221	P	Response to chemical stimulus	52	4909	3.40E-05	0.0088
GO:0009066	P	Aspartate family amino acid metabolic process	7	145	3.90E-05	0.0088
GO:0015698	P	Inorganic anion transport	7	144	3.80E-05	0.0088
GO:0000097	P	Sulfur amino acid biosynthetic process	5	67	7.50E-05	0.013
GO:0019748	P	Secondary metabolic process	20	1235	7.60E-05	0.013
GO:0009308	P	Amine metabolic process	19	1171	0.00011	0.017
GO:0050896	P	Response to stimulus	85	9863	0.00013	0.017
GO:0006555	P	Methionine metabolic process	5	88	0.00025	0.031
GO:0044106	P	Cellular amine metabolic process	15	890	0.00041	0.042
GO:0006520	P	Cellular amino acid metabolic process	15	890	0.00041	0.042
GO:0009755	P	Hormone-mediated signalling pathway	16	1003	0.00047	0.046
GO:0006820	P	Anion transport	8	297	0.00055	0.049
GO:0016491	F	Oxidoreductase activity	43	3249	9.10E-07	0.00041
GO:0009055	F	Electron carrier activity	24	1346	2.90E-06	0.00066
GO:0046906	F	Tetrapyrrole binding	17	819	1.40E-05	0.0018
GO:0020037	F	Heme binding	16	744	1.60E-05	0.0018
GO:0015103	F	Inorganic anion transmembrane transporter activity	7	154	5.70E-05	0.0051
GO:0005506	F	Iron ion binding	20	1268	0.00011	0.008
GO:0008509	F	Anion transmembrane transporter activity	8	251	0.00018	0.012
GO:0016209	F	Antioxidant activity	8	297	0.00055	0.03
GO:0022891	F	Substrate-specific transmembrane transporter activity	23	1842	0.00089	0.044
GO:0019825	F	Oxygen binding	9	409	0.001	0.045
GO:0016684	F	Oxidoreductase activity, acting on peroxide as acceptor	7	262	0.0013	0.046
GO:0042803	F	Protein homodimerization activity	7	268	0.0014	0.046
GO:0008289	F	Lipid binding	11	606	0.0014	0.046
GO:0004601	F	Peroxidase activity	7	262	0.0013	0.046

Ontology “P” indicates Biological Process, ontology “F” indicates Molecular Function, and ontology “C” indicates Cellular Component. “Number in input list” is the number of occurrence of the Gene Ontology term in the input list (262 genes). “Number in BG/Ref” is the observed occurrence of the Gene Ontology term in the *Populus trichocarpa* genome. *P*-value represent the statistical significance of the enrichement test and FDR is the false discovery rate set to 0.05.

**Table 4.** GO term enrichment analysis of the 27 differentially expressed genes with opposite trends in XU and XL (35° tilted vs. control).

GO term	Ontology	Description	Number in input list	Number in BG/Ref	*P*-value	FDR
GO:0044042	P	Glucan metabolic process	6	329	6.90E-08	1.60E-06
GO:0006073	P	Cellular glucan metabolic process	6	298	3.90E-08	1.60E-06
GO:0044264	P	Cellular polysaccharide metabolic process	6	457	4.60E-07	7.00E-06
GO:0005976	P	Polysaccharide metabolic process	6	639	3.10E-06	3.60E-05
GO:0044262	P	Cellular carbohydrate metabolic process	7	1093	5.10E-06	4.60E-05
GO:0005975	P	Carbohydrate metabolic process	7	2018	0.00025	0.0019
GO:0016758	F	Transferase activity, transferring hexosyl groups	5	707	8.90E-05	0.0033
GO:0016757	F	Transferase activity, transferring glycosyl groups	5	954	0.00036	0.0066
GO:0005506	F	Iron ion binding	5	1268	0.0013	0.016
GO:0005618	C	Cell wall	5	1051	0.00055	0.019
GO:0030312	C	External encapsulating structure	5	1207	0.001	0.019

Ontology “P” indicates Biological Process, ontology “F” indicates Molecular Function, and ontology “C” indicates Cellular Component. “Number in input list” is the number of occurrence of the Gene Ontology term in the input list (27 genes). “Number in BG/Ref” is the observed occurrence of the Gene Ontology term in the *Populus trichocarpa* genome. *P*-value represent the statistical significance of the enrichment test and FDR is the false discovery rate set to 0.05.

**Figure 4.**
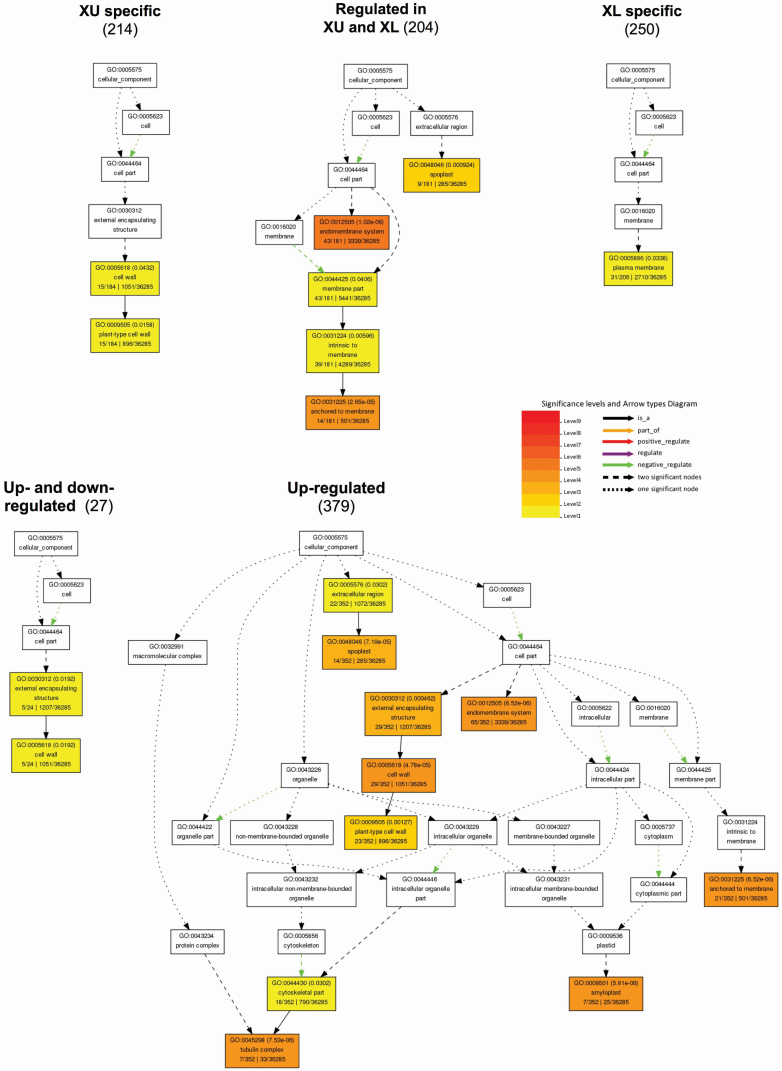
Graphical results of AgriGO cellular component terms analysis for differentially expressed genes put in different subgroups. Upper xylem specific (XU: 214 genes, 184 annotated), lower xylem specific (XL: 250 genes, 206 annotated), Genes modulated in both sides of xylem (204 genes, 181 annotated), Up-regulated genes (379 genes, 352 annotated), Up- and down-regulated (27 genes, 24 annotated). No significant cellular component term was found in the down-regulated subset. Relationships between terms are materialised by arrows described in the key. Coloured terms indicate a statistical signification: the more significant a term is, the redder the colour is. Term frequency found in *Populus trichocarpa* genome is indicated at the lower right corner and is used for statistical significance test.

By looking more precisely at CC terms impacted by the gravitational stimulation treatment, we discovered how the cells integrate the stimulus. Figure 4 is a graphical analysis of the CC term enrichment related to modulated genes. XU-specific genes appeared to be significantly linked to the ‘cell wall’ while the ‘plasma membrane’ was the only significant XL-specific CC. XU- and XL-modulated genes covered the ‘apoplast’ and the ‘endomembrane system’. When considering genes according to their expression tendency, no significant CC was identified for the 262 genes regulated downstream. The greatest variety of CC was found in upregulated genes including: ‘apoplast’, ‘cell wall’, ‘tubulin complex’, ‘amyloplast’, ‘endomembrane and membrane anchored’. For the 27 genes with upwards or downwards regulation on the xylem side, the ‘cell wall’ compartment was significantly impacted, in the same way as the specific XU genes (Fig. 4). Finally, it is interesting to note that among the genes expressed differently after gravistimulation, 36 were annotated as putative transcription factor (TF) **[see**  **Supporting Information—Table S4****]**.

### Exploration of candidate genes

**Figure 5.**
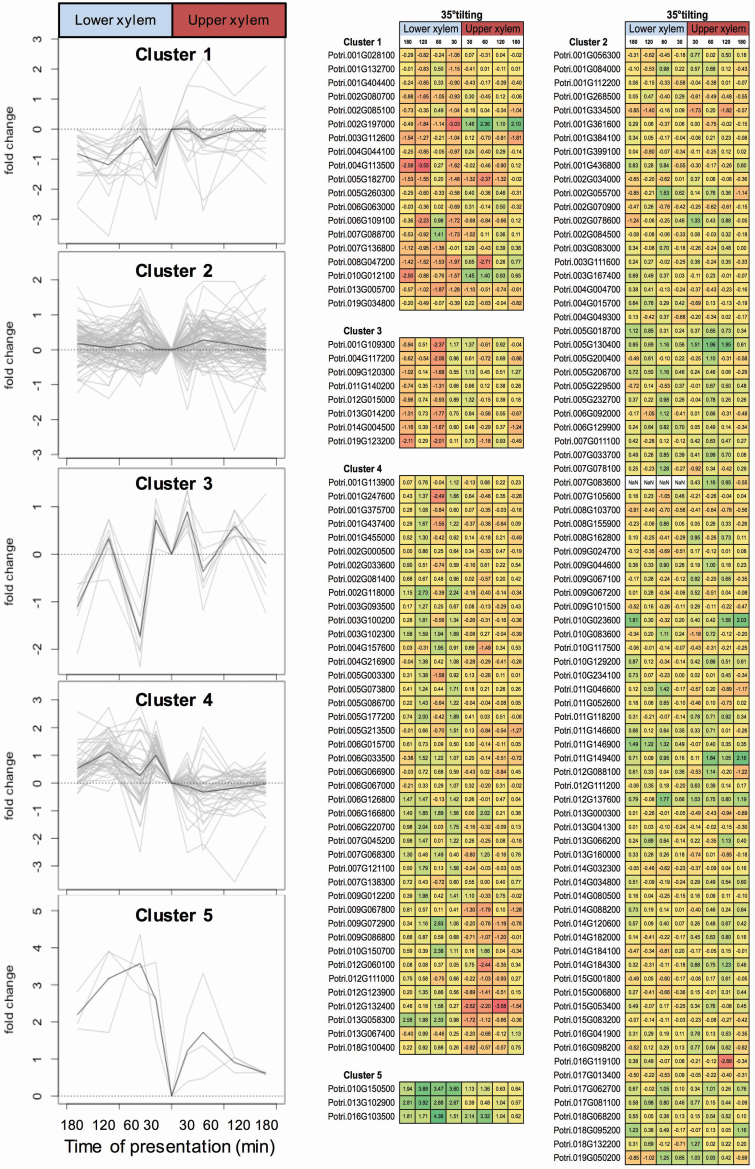
qPCR heatmap + clusters.

The experiments on DNA chips identified 668 genes with significant differential expression after 30 min of inclination at 35°. A subset (153 genes) was used to further investigate their involvement in gravitropic response by high-throughput quantitative PCR in a kinetic study (0, 30, 60, 120 and 180 min at 35°). As a prerequisite, we ensured the results of the biochips by reanalysing the expression of candidate genes found in the biochips experiment on five independent biological replicates. We found significant pairwise correlations between the two approaches (i.e. 0.67, 0.50, 0.71, 0.72, 0.67) allowing for a reliable interpretation of the data produced subsequently. We then performed a hierarchical grouping within the inclination expression data that led to the identification of five co-expression groups identified thereafter as Cluster #1 to Cluster #5 (Fig. 5).

Cluster #1 identified 19 genes mainly downregulated in XL after gravistimulation. A large bulk (8/19) concerned the metabolism of sugars (glycosyl groups and/or carbohydrates), whereas another group of five genes had something to do with auxin and/or ethylene responses and/or metabolism. Lastly, four genes were involved in transmembrane transport. Cluster #2 comprised the highest number of genes (81) and a wide panel of metabolic processes. Using a KEGG enrichment analysis, three major pathways were brought forward including photosystem II oxygen-evolving enhancer protein 1 (i.e. OEE), the SAUR protein family (i.e. auxin responsive genes) and β-tubulin (i.e. cytoskeleton). Briefly, cell redox players, putative mechanical sensors, various kinases, several brassinoid, ethylene- and auxin-related genes and TFs were well-represented in that cluster. It is also in Cluster #2 that we found genes already known to be involved in lignin synthesis and cell wall modification. Cluster #3 identified three Fasciclin-like arabinoglactan genes, expression of which was bimodal in XU and XL reaching two maxima at 60 and 180 min after gravistimulation. Cluster #4 was the second largest cluster with 42 genes over-expressed in XL and whose expression was not modified in XU. Functional annotations highlighted phenylpropanoid, flavonoid and terpenoid biosynthesis. Of note within that cluster was the significant BP enrichment in ‘transmembrane transport’ especially concerning sugars. ‘Lipid metabolic process’ was the second biological process pinpointed. Finally, Cluster #5 was the smallest cluster identified with only three co-expressed genes upregulated in both XL and XU. Functional annotations suggested that oxidative stress and oligopeptide mobilization were the processes concerned.

## Discussion

### The experimental device allowed to identify genes regulated by inclination

This study aimed to better understand the molecular processes that occur in the secondary xylem of the woody stem after short-term tilting while separating the phototropic effect from gravitrophic stimulation. To do so, we used an innovative experimental design (Coutand *et al.* 2019) to identify a list of differentially expressed genes specifically responding to gravitropism without interference with the phototropic response. Stem staking in our condition was shown to be effective in preventing stem deformation as assessed by the absence of induction of PtaZFP2, a well-characterized reporter gene for stem bending in poplar (Martin *et al.* 2009). It is interesting to note that we were able to record very early molecular responses, whereas previous studies, which did not use our innovative device and are therefore difficult to compare, found significant differential gene expression only after 8 h of gravistimulation (Zinkgraft *et al*. 2018). It is difficult to say whether this difference is due to the distinctive statistical power of the studies or whether it is due to other factors. The idea that an early molecular adjustment could occur in the gravitropic response is however supported by our earlier observation that changes in the poplar stem proteome were recorded as early as 45 min after tilting (Azri *et al.* 2009).

### Genes supporting cell wall reorganization are modulated in ‘opposite and tension wood’ forming zones

Among the many gene classes identified in this study, we have chosen to first focus on the genes involved in cell wall formation and modification. The cell component ‘cell wall’ was indeed one of the CCs of interest and this choice was strongly supported by the biological process terms that were significantly brought forward in our analysis including: ‘cell wall biogenesis’, ‘carbohydrate metabolic process’ and ‘catabolic lignin process’. Within that compartment, our experimental set-up allowed the identification of genes involved in wood formation specifically regulated by inclination independently of the phototropic stimulation response. It was interesting to note that as early as after 30 min of gravistimulation modulation of gene expression was recorded on both sides of the inclined stem (Fig. 3), thus concerning the whole secondary xylem. From the analysis of the GO enrichment (Fig. 4), it appears that the changes mainly affect the cell wall compartment on the upper surface, which corresponds to the installation of G-layer fibres (Jourez *et al.* 2001). For the lower xylem, the plasma membrane is the significant cellular component affected, which could indicate the importance of control between adjacent compartments. Some genes, encoding mainly TFs involved in the formation of the secondary cell wall, appear to be regulated in both XU and XL. This is the case with Potri.001G112200, a KNAT7 homologue (class II KNOX gene) that has been shown elsewhere to be a key negative regulator of secondary cell wall formation (Li *et al.* 2012). Bhargava *et al.* (2013) suggested that KNAT7 could be involved in the suppression of lignin biosynthesis in stems. The analysis of promoters of genes encoding such TF sensitive to gravitational stimulation could provide a good opportunity to decipher molecular events upstream.

Rose *et al.* (2004) suggested that cell wall remodelling involves a complex interplay of enzymatic and non-enzymatic oxidative and hydrolytic changes. In agreement with this we found in Cluster #2 several redox-regulating players including: four oxygen-evolving enhancer proteins, a glutathione *S*-transferase, thioredoxin h, three 2OG-Fe(II) oxygenases and a peroxydase. We also found within that same cluster eight carbohydrate active enzymes (CAZymes), four glycosyltransferases (GTs) and four glycoside hydrolases (GHs) that could participate in modulating the composition and structure of gravistimulated xylem cell wall. The fact that they were all clustered together supports a synergistic effect on cell wall properties as suggested earlier by Tenhaken (2015). In addition, the observation that these genes also clustered with genes involved in regulation of cell expansion (namely Potri.009G067100, Potri.009G067200 and Potri.008G16280) suggests that they all may be mobilized during cell wall modifications accompanying cell expansion.

A well-established process is that of the reprogrammation of sugar metabolism when normal wood is compared to TW (Mizrachi *et al*. 2014). Several reports (Lafarguette *et al.* 2004; Paux *et al.* 2005; Andersson-Gunneras *et al*. 2006) showed that in TW (upper side), the upregulation of cellulose synthase activity was responsible for the production of the so-called gelatinous layer (G-layer). In our experimental setting, reprogrammation of sugar metabolism appears to occur on both sides of the stem shortly after gravistimulation as evidenced by the increased expression of two genes encoding cellulose synthases in XL and XU (CESA4 Potri.004G059600, Potri.002G257900). In addition, the expression of two cellulose synthase-like genes (CSL, β-1,4-mannan synthases) was downregulated in the two xylem sides. In poplar it was shown that Fasciclin-like arabinogalactan proteins (FLA) were induced after gravistimulation and thought to participate in TW G-layer synthesis (Lafarguette *et al.* 2004; Fagerstedt *et al.* 2014; MacMillan *et al.* 2015; Wang *et al.* 2015). We show here that a bulk of 10 FLA were upregulated after 30 min of gravistimulation. Cluster #1 also brought forward genes involved in sugar transport/removal/hydrolysis (Potri.005G260300, and the CAZymes: Potri.001G028100, Potri.004G044100, Potri.004G113500, Potri.002G080700, Potri.003G112600) suggesting that sugar repartition is modified in xylem after inclination.

In agreement with the observation made by Villalobos *et al.* (2012) that ‘opposite wood’ (lower side) has a greater lignin content than TW we found four upregulated LIM TFs (Potri.002G157300, Potri.002G118000, Potri.009G087200, Potri.014G080900) that were suspected to play a role in lignin biosynthesis (Chen *et al.* 2015). Our analysis also brought forward genes involved in monolignols production such as phenylalanine ammonia-lyase (PtPAL1.2, Potri.006G126800) expression of which was repressed in XL and, 4-coumarate:CoA ligase (4CL, Potri.003G188500) repressed in XU along with caffeoyl-CoA *O*-methyltransferase 1 (CCoAOMT1, Potri.009G099800). In addition, Potri.016G112100, a laccase 1 homolog was found upregulated in XU although it is not yet consensually admitted that it participates in the lignification process (Gavnholt and Larsen 2002; Berthet *et al.* 2012). Consistent with the idea that TW has a lower lignin content we found that PtSAMS1 (Potri.013G004100), a *S*-adenosylmethionine synthetase (SAMS) suspected to play a central role in lignin biosynthesis (Vander Mijnsbrugge *et al.* 2000; Shen *et al.* 2002), was inhibited in XU.

Finally, we also found that two sequences (Potri.011G006800 and Potri.004G009600) encoding two T-proteins of the glycine decarboxylase complex (GDC) participating in serine biosynthesis were downregulated in XU. This is in line with the suggestion that GDC may allow woody perennials to cope with C1 demands of lignification (Rajinikanth *et al.* 2007). It is also consistent with the report of GDC involvement in the context of compression wood formation (Villalobos *et al.* 2012).

### Impact of gravistimulation on wood cell expansion

Some of the significant BP, MF and CC terms brought forward by the present study strongly suggest that wood cell expansion is a process impacted by gravistimulation. This is in agreement with a growing number of studies linking gibberellins and TW (Eriksson *et al.* 2000; Mauriat and Moritz 2009; Nugroho *et al.* 2012, 2013; for a review, see Felten and Sundberg 2013). The prevailing view, as reviewed recently by Ruelle (2014), is that in poplar TW fibres are longer and thinner when compared to that in ‘opposite wood’, suggesting complex regulation of cell expansion processes. In agreement with these observations, we found that Potri.012G132400 and Potri.015G134600, two active gibberellins (GA20OX) coding genes that have been linked elsewhere with cell expansion were modulated (Israelsson *et al.* 2005). Besides gibberellins (GA), other phytohormones are known to play a role in cell expansion. In particular, brassinosteroids (BR) were shown to be involved in stem reorientation and cell elongation (Bienert *et al.* 2012; Vandenbussche *et al.* 2013). Interestingly, we found that a homolog to BRI1 (brassinosteroid-insentitive1, an *A. thaliana* cell surface LRR-S/T kinase receptor for BR) (here identified as Potri.007G078100), and a serine carboxypeptidase (SCP) homolog to brassinosteroid insensitive1 suppressor (BRS1) (here identified as Potri.011G046600) were found modulated within Cluster #2 in response to gravistimulation. Of course, we cannot rule out the hypothesis that the listed genes involved in detection/response to GA and BR have a role in the regulation of cell differentiation and secondary cell wall biogenesis as suggested by Du *et al*. (2019). In addition, GA may regulate key genes involved in BR response (Gerttula *et al.* 2015).

As cell expansion is regulated by the plasticity of the primary cell wall (Cosgrove 2005) it was consistent to see that after our stimuli several genes encoding proteins involved in loosening tension between primary cell wall constituents were found modulated, including expansin, expansin-like and pectin xyloglucans modifier genes (Potri.007G083400, Potri.010G167200, Potri.013G134300, Potri.002G060500, Potri.013G005700, Potri.014G115000, Potri.018G084300).

At last, supporting the involvement of cell expansion in response to gravistimulation we recorded the upregulation of Potri004G117200 in XL. This sequence is a homolog of the *A. thaliana* COBRA protein, an extracellular glycosyl-phosphatidyl inositol-anchored protein required for polarized cell expansion through cellulose microfibril orientation (Schindelman *et al.* 2001; Roudier *et al.* 2005).

### Could concerted regulations of flavonoid and phosphoinositides pathways occur after gravistimulation?

Following gravistimulation, signal transduction leads to complex biochemical and genomic responses at the subcellular level. In Cluster #4, we observed the co-expression of genes involved in phenylpropanoid biosynthesis (PAL1—Potri.006G126800) and flavonoid (Potri.002G033600, Potri.003G100200) pathways. The TF LIM1 (Potri.002G118000) is also found in this cluster which agrees with Chen *et al.* (2015) who have found co-expression in ‘opposite wood’ of several LIM TFs with PAL genes. Several studies suggest that the synthesis or deposition of flavonoids regulates auxin transport during gravitropic responses (Geisler *et al.* 2005; Buer *et al.* 2007, 2013; Santelia *et al.* 2008; Lewis *et al.* 2011). Gayomba *et al.* (2016) demonstrated their role in auxin transport inhibition and antioxidant activity in *Arabidopsis*.

Furthermore, it is known that phosphoinositides regulate vesicle trafficking (for a review, see Krishnamoorthy *et al.* 2014) and auxin-dependent development (Tejos *et al.* 2014). Phosphatidyl inositol transfer protein *SEC14* (Potri.004G157600) is co-expressed in Cluster #4 with CLATHRIN assembly protein (Potri.006G066900) indicating the regulation of vesicle trafficking. Lipid metabolism is also regulated (Potri.006G166800, Potri.012G12390).

In this context we hypothesize that concerted regulations of the flavonoid and phosphoinositides pathways led to auxin modulation after gravistimulation, probably through vesicle trafficking.

In the research areas of nutrition and dietetics, it has been shown that flavonoids exert modulating actions on the cellular system through direct action on various signalling pathways such as those of phosphoinositide 3-kinase, and protein kinase C (Mansuri *et al.* 2014).

### What about cytoskeleton involvement in xylem following gravistimulation?

On the understanding that the involvement of the cytoskeleton following shoot gravistimulation is still an unsolved question (Song *et al.* 2019), we noticed that CC terms such as ‘membrane and endomembrane systems’, ‘cell wall’, ‘apoplast’, ‘amyloplast’, ‘vacuole’ and ‘tubulin complex’ were affected in our gravistimulation experiment. The observation that the expression of tubulin genes, as well as that of an actin-depolymerizing factor (Potri.001G106200) and a F-actin capping protein alpha subunit (Potri.013G017000) is modulated suggests that modification of the cell cytoskeleton is partly a response to gravistimulation as suggested earlier by Nick (2011). It was shown already in *Arabidopsis* stem that the actin microfilaments may be considered as negative regulators of the gravitropic response considering their interaction with the amyloplasts through the link to SGR9 (Kolesnikov *et al.* 2016). Song *et al.* (2019) highlighted the role of the actin-bonding protein Rice Morphology Determinant (RMD) in the regulation of the gravitropic response of the light-grown shoots. In different studies, microtubules were also shown to participate in gravitropism mainly via cellulose deposition in xylem (Fagerstedt *et al.* 2014; Araniti *et al.* 2016).

To conclude, our experimental isotropic light experimental set-up allowing the strict analysis of gravitropic stimulation of trees not polluted by the phototropic response highlighted a list of 668 xylem-regulated genes involved in early gravitropic response. Some of the genes pinpointed here were already reported to participate in gravity response confirming the pertinence of our approach and attesting that they were indeed related to the gravity stimuli and not to another confounding factor (phototropic response, stem bending response). Interestingly, more than a third of these genes are still not annotated for gene function. We consider that this particular gene subset, especially those still not annotated, constitutes a good starting point to expand our current knowledge on gravitropism. This work opens new research prospects towards the understanding of events triggering ‘reaction wood’ formation.

## Supporting Information

The following additional information is available in the online version of this article— 

**Table S1.** List of qPCR primers.

**Table S2.** List of the 2355 differentially expressed genes based on poplar annotation V1.

**Table S3.** List of the 668 differentially expressed genes based on poplar annotation V3.

**Table S4.** List of transcription factors.

**Figure S1.** Correlation analysis of microarray data.

**Figure S2.** High-resolution version of Fig. 3 with gene model names.

**Figure S3.** GO term enrichment of Biological Processes (BP), Molecular Functions (MF) and Cellular Components (CC) of the 668 differentially expressed genes in XU and XL.

plaa071_suppl_Supplementary_Table_S1

plaa071_suppl_Supplementary_Table_S2

plaa071_suppl_Supplementary_Table_S3

plaa071_suppl_Supplementary_Table_S4

plaa071_suppl_Supplementary_Figure_S1

plaa071_suppl_Supplementary_Figure_S2

plaa071_suppl_Supplementary_Figure_S3

## Notes

**Form & Function. Chief Editor:** Kate McCulloh
