# Supplementary figures and images for: Early transcriptional response to gravistimulation in poplar without phototropic confounding factors
Source: AoB Plants. 2020 Dec 31;13(1):plaa071. doi: 10.1093/aobpla/plaa071 (PMC7850117; doi:10.1093/aobpla/plaa071)

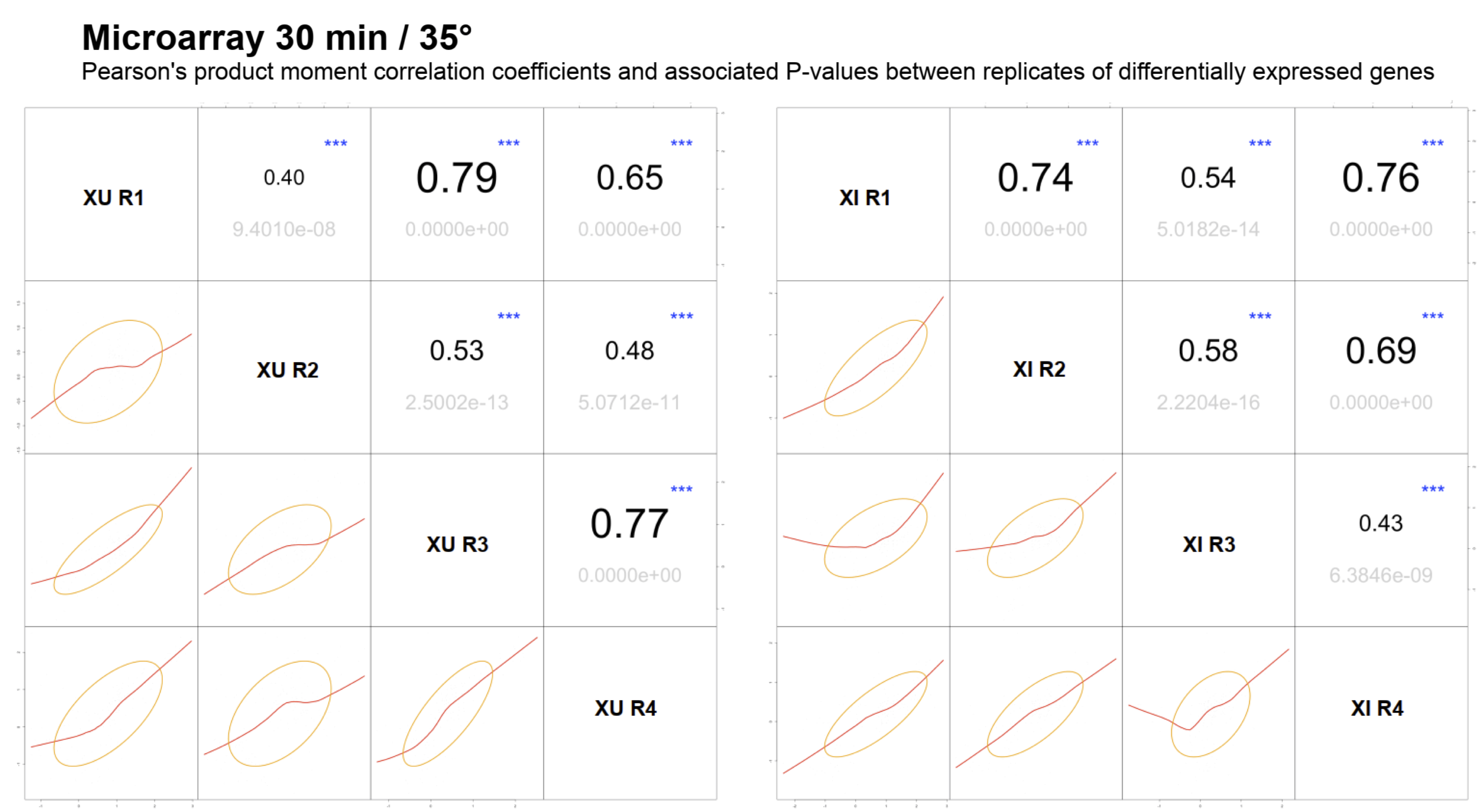

Supplement: plaa071_suppl_Supplementary_Figure_S1 [file plaa071_suppl_supplementary_figure_s1.jpeg]

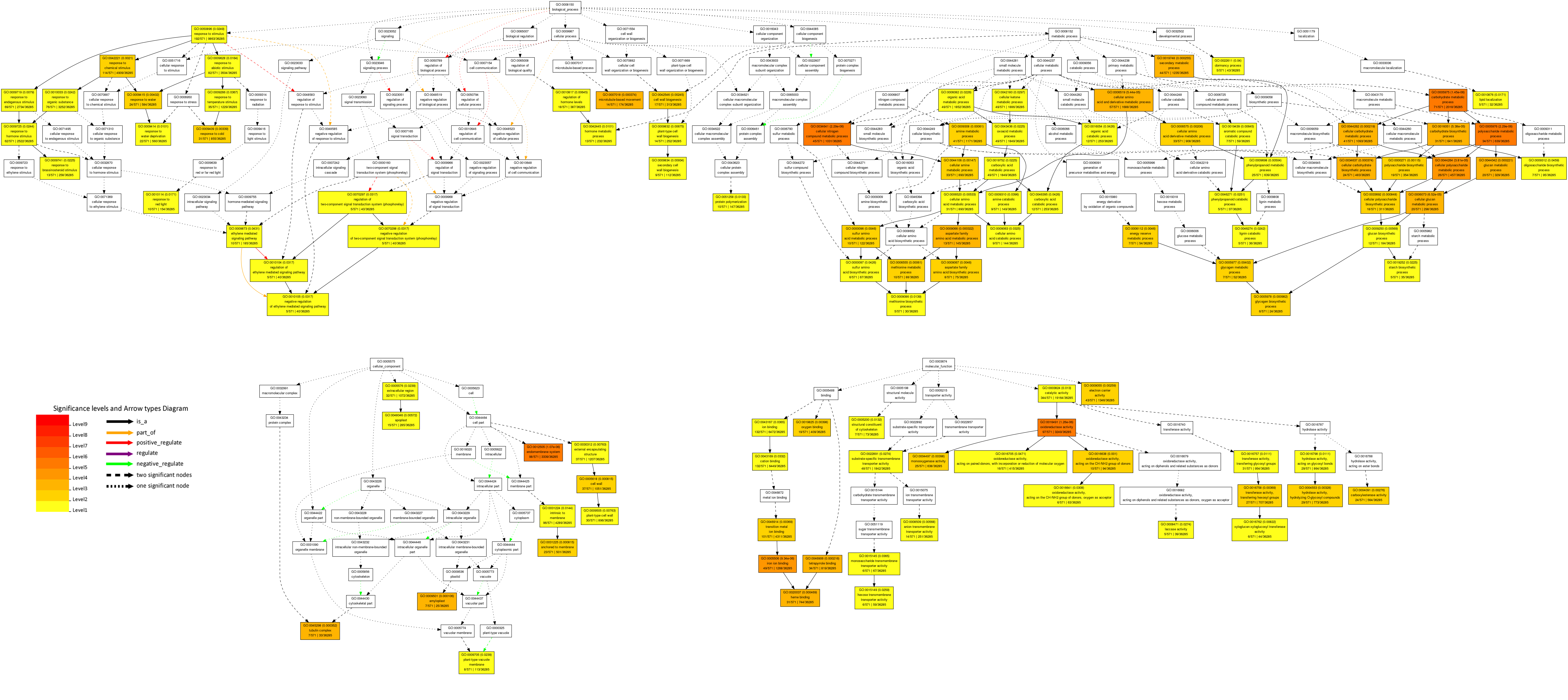

Supplement: plaa071_suppl_Supplementary_Figure_S3 [file plaa071_suppl_supplementary_figure_s3.jpeg]
